# Unpacking the effects of brand authenticity on consumer trust and loyalty in nutrition: a psychological perspective

**DOI:** 10.3389/fnut.2025.1731800

**Published:** 2026-01-09

**Authors:** Mingkun Lyu, Shikai Wang, Jinwei Zhang

**Affiliations:** Department of Business Administration, Silla University, Busan, Republic of Korea

**Keywords:** brand authenticity, brand loyalty, consumer trust, emotional attachment, food availability, nutrition branding, perceived sincerity

## Abstract

**Background:**

Brand authenticity plays a crucial role in shaping consumer evaluations in nutrition markets, where product claims are difficult to verify and consumers rely heavily on trust.

**Objective:**

The research investigates the influence of perceived brand authenticity on consumer trust and loyalty, and also examines the mediating role of perceived sincerity, self-congruence, and emotional attachment in that connection.

**Method:**

Using survey data from 300 consumers, the measurement and structural models were validated through Confirmatory Factor Analysis (CFA) and Structural Equation Modelling (SEM) with bootstrapped indirect effects.

**Results:**

The results demonstrate that brand authenticity significantly enhances trust (*β* = 0.54, *p* < 0.001), and that trust strongly predicts loyalty (*β* = 0.47, *p* < 0.001). The indirect effect of authenticity on loyalty through trust was significant, indicating full mediation. Additional indirect pathways by sincerity, self-congruence, and emotional attachment were also supported.

**Conclusion:**

These findings clarify the cognitive and affective mechanisms linking brand authenticity to trust and loyalty in nutrition branding, offering strategic insights for managers aiming to strengthen long-term consumer relationships. In this way, the study addresses the trust and authenticity nexus during nutrition branding and demonstrates how to credibly and systematically approach brand storytelling in critical health issues.

## Graphical abstract

**Figure:**
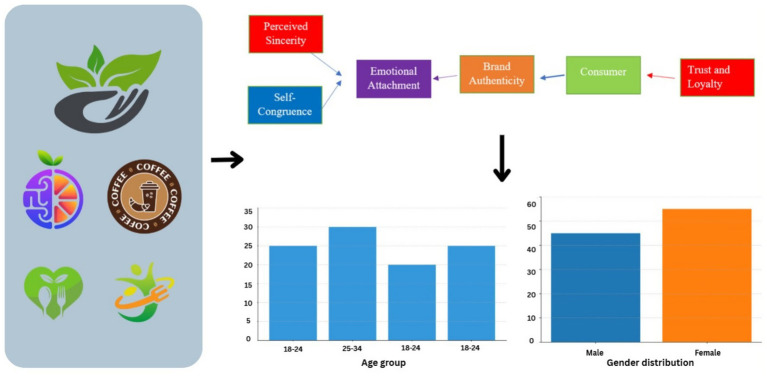

## 1 Introduction

In recent years, consumers have become increasingly health-aware, and nutrition and wellness markets have expanded rapidly. In these markets, consumers frequently face information asymmetries: product health claims often involve credence attributes that cannot be fully perceived authenticity to reduce uncertainty and make purchase decisions. Understanding how brand authenticity translates into consumer trust and, ultimately, loyalty is therefore important for both theory and practice in nutrition branding.

**Figure 1:**
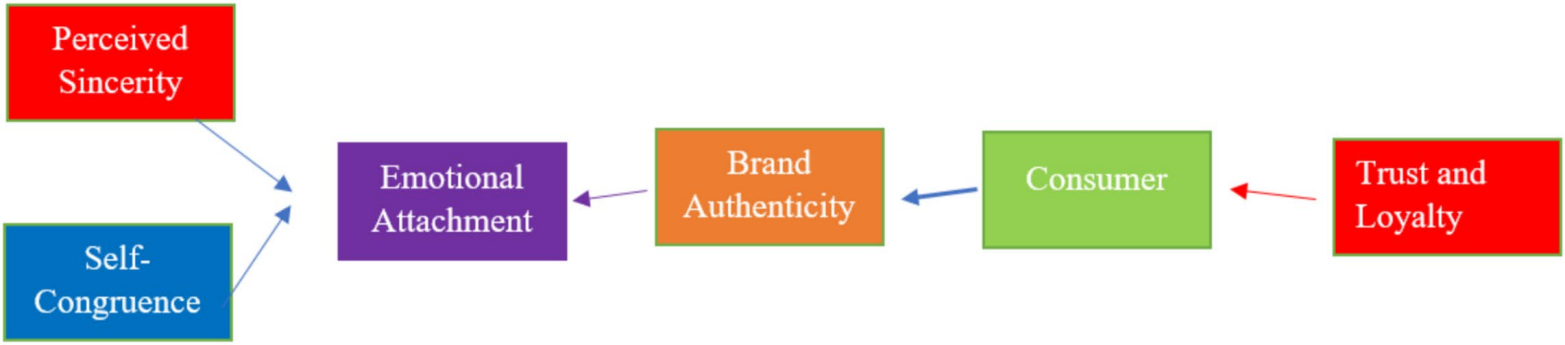
Proposed authenticity-trust–loyalty framework (nutrition branding).

Prior consumer-behavior research has established broad links between brand authenticity, trust, and loyalty, but many studies treat authenticity as a single undifferentiated predictor, less is known about the mechanisms through which authenticity operated (for example, cognitive self-congruity, perceived sincerity, and affective attachment) and how these mechanisms function in credence/health contexts, where perceived risk and verification costs are higher. To address this gap, we integrate Self-Determination Theory (SDT), Social Identity Theory (SIT), and affects trust and loyalty in the nutrition domain (1). The marketplace is changing rapidly and so are the attitudes of informed and skeptical consumers who have an acute awareness of the health and nutrition. Figure 1 presents the conceptual framework that links perceived authenticity, proximal mechanisms (sincerity, self-congruence, emotional attachment), trust and loyalty; this model guides the hypothesis and empirical tests in the research.

Authentic brands are those that are perceived to possess a true ethos, operational transparency, and aligned practices and messaging. Within the context of nutritional branding, this type of authenticity is especially important (2).

Consumers often develop strong emotional and psychological attachments to nutrition products, particularly because these products are closely associated with health and well-being. A perceived authenticity of a brand brings an impression of security and trust which makes consumers assume that whatever the brand claims is positive and worthwhile. The psychological foundation on which the loyalty of consumers to such brands is explained is the so-called psychological connection of authenticity and trust (3).

Although these psychological theories are insightful, there continues to be a significant gap in understanding how brand authenticity affects trust and loyalty in the context of nutrition. Most of the existing work has focused on authenticity in branding and consumer behavior more generally, with little focus on nutrition branding. Most critically, there is no research for an integrative model that connects authenticity, trust, and loyalty in a unified framework. This gap emphasizes the need to understand how perceptions of brand authenticity are transformed into psychological constructs such as trust, and how these constructs impact loyalty to a brand in the context of nutrition (4).

Although recent work has examined authenticity and trust in consumer settings, much of that literature treats authenticity as a broad predictor without differentiating mechanisms or contextual moderates (5). This paper differs in three important ways. First, we assign specific psychological mechanisms (motivational/ internalization, identity signaling, and cognitive self-congruity) to model. Second, we examine authenticity in a credence/health domain (nutrition) where verification is difficult and perceived risk is elevated, a context that amplifies some mechanisms (e.g., uncertainty reduction by self-congruity) compared with standard consumer categories. Third, we provide empirical mediation evidence (SEM with bootstrapped indirect tests, *N* = 300) and propose boundary conditions (perceived health risk; product credence level) that quality when each mechanism is most influential. We therefore present an incremental but theory-driven advance that refines rather than repeats findings from broader consumer behavior research.

### 1.1 Research objectives and hypothesis

The study addresses the following objectives, to examine how perceived brand authenticity influences consumer trust toward nutrition brands. To assess whether trust mediated the relationship between brand authenticity and consumer loyalty. To identify proximal mechanisms (perceived sincerity, self-congruence, emotional attachment) that explain how authenticity generates trust.

We analyzed cross-sectional survey data (*N* = 300) using confirmatory factor analysis (CFA) and structural equation modelling (SEM) with bootstrapped indirect effects. Our contributions are threefold: (a) we assign specific mechanisms to established theories and test them jointly in a credence context; (b) we provide empirical mediation evidence clarifying the process from authenticity to loyalty by trust; and (c) we identify moderators and managerial levers relevant for nutrition brads (e.g., transparent evidence, identity cues, and third-party verification).

Based on theory and prior research, we test the following hypothesis:

*H1*: Perceived brand authenticity is positively associated with consumer trust.

*H2*: Consumer trust is positively associated with brand loyalty.

*H3*: Trust mediated the relationship between authenticity and loyalty (i.e., authenticity → trust → loyalty).

*H4*: Perceived sincerity and self-congruence operate as proximal mediators of the authenticity → trust link; emotional attachment acts as an affective mediator that supports trust’s effects on loyalty.

The remainder of the paper proceeds as follows, section 2 develops the conceptual framework and hypothesis, section 3 describes the sample and methods, section 4 reports measurement and structural results, and section 5 discusses implications, and section 6 describes conclusion and future research.

## 2 Conceptual framework

This section integrates three complementary psychological perspectives, Self-Determination Theory (SDT), Social Identity Theory (SIT), and Self-Congruity Theory, to explain how perceived brand authenticity translates into consumer trust and loyalty in the nutrition (credence) domain. In brief: authenticity reduces cognitive uncertainty (self-congruity), supports internalization of brand values (SDT), and enables identity signaling (SIT); these mechanisms operate through proximal mediators (perceived sincerity, self-congruence, emotional attachment) to increase trust, which turn drives loyalty (6). This section uses various psychological theories to bridge the gap on the interrelationship between brand authenticity, trust, and loyalty and examines the relevance of these elements in the nutrition sector.

### 2.1 Theoretical foundation: bridging the gap on authenticity, trust and loyalty

Several theories explain why authenticity fosters trust and loyalty. Self-Cognitive Theory predicts that consumers prefer brands that match their self-concept; congruence reduces uncertainty and strengthens loyalty (4). SIT suggests that brands that signal group membership encourage social validation and sustained attachment (7). In the nutrition domain, these mechanisms are amplified because product claims are often credence-based and health-relevant, which increases the need for identity and cognitive shortcuts. Finally, the Attitude-behavior Consistency perspective links favorable attitudes to repeat purchase behavior, showing how trust can translate into actual loyalty. If a consumer has trust towards a nutrition brand because of its authenticity, there is a greater likelihood of loyal purchase behavior to take place (8).

Brand authenticity rests on clearly communicated practices (transparency, ethical sourcing, and consistent messaging) that align with consumer’s self-concepts and values. In nutrition a credence and high-stakes domain authenticity signals more than corporate virtue: it reduces uncertainty about health claims and functions as a trust cue that supports repeated purchase behavior. Thus, authenticity in nutrition operates through both cognitive (uncertainty reduction, self-congruity) and affective (identity alignment, internalized motivation) channels. These dual pathways motivate the hypothesis in this paper (9).

### 2.2 Self-determination theory (SDT): authenticity and intrinsic motivation

SDT suggests authenticity facilitates internalization of brand values: when nutrition brands provide credible evidence and transparent messaging, consumers internalize those values as compatible with their own, increasing autonomous motivation to rely on the brands claims. In credence domains, this internalization reduces reliance on external cues (e.g., price) and strengthens trust, particularly when health stakes are perceives as high. Authentic brands are viewed as honest and transparent, thus, fulfilling the consumer’s psychological autonomy and self-congruence. When consumers perceive that a brand is authentically aligned with their personal values, they are more likely to experience motivation to engage with the brand, thus, increasing consumer satisfaction and fulfillment. Therefore, brand motivation as an underlying factor of consumer motivation is driven by authenticity. This motivation determines the consumer’s emotional bond to the brand (10). As an example, in nutrition branding, SDT hypothesizes that the more clearly a protein supplement or a functional food brand in the industry shares third-party laboratory test results, source of ingredients, and health claims supported by evidence, the higher the rate at which the brand will internalize its commitment to quality and consumer well-being. As a result, the relationship between the consumers and the brand is built on the perceived credibility and expertise.

### 2.3 Social identity theory (SIT): belongingness and identity alignment

SIT emphasizes how brands become identity signals. Authentic nutrition brands enable consumers to express health-oriented identities and again social reinforcement from group membership; this social validation strengthens affective attachment and trust. The more a brands cues align with an identity script (e.g., organic, evidence-driven), the greater its capacity to generate loyalty by social mechanisms. In the world of nutrition, ‘health’ or ‘organic’ brand communities are widely supported, in addition to the other sociocultural popular communities. Belonging to such social groups, consumers are attracted to authentic brands that ‘give’ and enhance the consumer’s feeling of belonging such social communities. They consequently, grow their bonds with the particular nutrition brand. The level of perceived authenticity of the nutrition brand causes the consumers to become more ‘health oriented.’ It also indirectly extends their trust and loyalty towards the brand. It self-perpetuates the cycle of trust and loyalty (11). The consumers are more inclined to engage with the brands that help foster their group identity. Thus, reinforcing the brand’s authenticity.

In the context of nutrition, SIT manifests when consumers associate themselves with a brand that symbolizes health-focused identities, such as being labelled as organic, plant-based, or evidence-based, which represent a shared lifestyle. In other words, consumers who consider themselves to be fitness-oriented or health-conscious might build more trust and loyalty to the brands of nutrition that publicly involve themselves in sustainability campaigns and community health programs, or use certified organic labelling, as these visual cues enhance group membership and social recognition.

### 2.4 Cognitive–affective–behavioral model

The Cognitive–Affective–Behavioral Model articulates how authenticity impacts trust and loyalty by different psychological pathways. Authenticity first affects the cognition domain when consumers hold beliefs about a brand’s reliability and transparency. When a brand is viewed as authentic, it is seen as trustworthy which results in positive cognitive appraisals.

The emotional responses as discussed in the ‘affective pathway’ touches on how authenticity evokes attachment, warmth, and reassurance in a positive manner (8). In the nutrition domain, these emotions run high as consumers entrust these brands with their health and well-being. This emotional connection ultimately enhances consumer satisfaction and increases loyalty. The proposed model suggested that the first step that consumers take in the process of authenticity evaluation is to filter such cues as label transparency, traceability of ingredients, regulatory certifications, and so forth. Such assessments, in their turn, lead to affective reactions, such as reassurance and emotional bonding, especially in cases of high-risk health outcomes. Repeated positive experiences over time will tend to strengthen the bond to a specific behavior, as the consistent repurchase of a reliable nutrition brand even in the presence of lower-cost or more intensively promoted substitute brands.

### 2.5 Theoretical integration and novel contributions

While SDT, SIT and Self-Congruity Theory each offer useful perspectives on consumer-brand relations, their combined explanatory power is especially valuable for nutrition branding, which is characterized by (a) credence attributes (consumers cannot fully verify health claims before use), (b) heightened perceived risk (direct consequences for health), and (c) identity-relevant consumption (health choices signal values and group membership). Integrating the three theories clarifies distinct mechanisms by which authenticity translates into trust and then into loyalty in this context.

SDT explains how authenticity satisfies intrinsic needs for autonomy and coherence: when nutrition brands signal genuine concern for consumer welfare (transparent sourcing, evidence-based claims), consumers experience internalized motivation to accept the brand increasing willingness to rely on its health claims and thereby strengthening trust.

SIT highlights how authentic nutrition brands enable consumers to signal membership in health-oriented communities. Where product choices are identify-relevant, perceived congruence between self and brand increases affective commitment and social reinforcement of brand trust.

Self-congruity captures the cognitive match between consumer’s self-concept and brand attributes. In credence domains, perceived congruence reduces uncertainty (a cognitive shortcut), lowering perceived risk and facilitating reliance on the brands trust. Integrating these mechanisms produces two key theoretical advances. First, it clarifies a sequential-cum-multi-mechanistic path: authenticity → (perceived sincerity / self-congruence) → internalized motivation and identity alignment → trust → loyalty. Second, it identifies contextual moderators unique to nutrition branding → notably perceived health risk and product credence level, that amplify or attenuate pathways. These refinements move beyond a generic recitation of theories by: (a) assigning each theory a specific mechanism in the model, (b) explaining why those mechanisms are particularly potent for nutrition brands, and (c) generating testable boundary conditions that advance theory and guide future research.

SDT, SIT, and self-congruity explain complementary mechanisms in nutrition branding: SDT for internalization/ motivation, SIT for social-identity signaling, and self-congruity for cognitive uncertainty reduction. In this credence context, assigning each theory to a distinct mechanism clarifies how authenticity operates and identifies moderators (perceived health risk; product credence) that alter pathway strength.

### 2.6 Study contributions and boundaries

This study presents four highly focused, theory-enhancing contributions situated within clearly defined boundaries, thus extending rather than replacing the corpus of research on consumer behavior. First, it assigns each theoretical lens to a distinct underlying mechanism, SDT to internalization and autonomous motivation, SIT to social and identity signaling, and self-congruity theory to cognitive uncertainty reduction and empirically examines their relative influence in a nutrition-branding context. Second, the research is fundamentally concerned with credence-based nutrition and health products, in which the scale of information void and the perceived health stakes are large, it becomes necessary to encourage consumers to resort to brief psychological shortcuts and signals of authenticity. Third, they use an SEM based on bootstrapped indirect effects (*N* = 300) to test the main mediators; perceived sincerity, self-congruence, and emotional attachment, and warn of possible moderators such as perceived health risk and product credence, which can reinforce or weaken any one of these paths. Lastly, they sketch actual management levers, including visible evidence, identity-congruent communications, and third-party validation, mentioning that the use of these strategies may not be effective across the board based on consumer characteristics and circumstances.

### 2.7 The function of consumer trust within the primary framework

The emotional attachment is bridged by the trust mechanism. It operates parallel to the first factor, which is trust. Brand loyalty is complicated by the endorsement of a brand. An endorsement is not the same as advocacy, as endorsement implies frequent brand purchase. Advocates purchase a brand only occasionally.

The word endorse requires adequate explanation. Specifically, the brand need not only to meet the expectations of the consumers but to also supersede their expectations. This carries a great emotional appeal. It is an emotional appeal that makes life more meaningful (12). This is essential in the nutrition industry. Authenticity of a nutrition brand does not guarantee repeat purchases. It retains consumers whose trust is enjoyed. Trust is the validation of brand loyalty (13).

Trust fosters both emotional and cognitive brand loyalty. The adjectives cognitive and emotional describe the same type of brand loyalty. An endorsement is sufficient but not necessary to foster trust. Therefore, trust is needed in order to establish authentic behaviors into appropriate, repeat purchase loyalty.

Brand loyalty emerges as both behavioral and attitudinal, and is a result of sustained authenticity and trust, with attitudinal loyalty defined as having a positive brand disposition, with attitudinal behavioral loyalty being repeated purchase and engagement with a brand. In the context of nutrition, brand authenticity and trust are likely to attitudinally and behaviorally brand loyal, and thus, the construct is a predictor of long-term brand success (14).

### 2.8 Gaps in the literature

To clarify the incremental and context-specific contributions of the present study, Table 1 compares the key points of previous consumer-behavior research with the new theoretical and empirical developments we are introducing. It demonstrates the similarities and differences between our research and related studies by highlighting contextual specificity, the theoretical mechanisms we have attributed to them, and the boundary conditions that are evidently related to nutrition branding.

**Table 1:** Comparison of the prior research and the current study.

Aspect	Prior research	Current research
Context	General consumer goods and services	Nutrition branding as a credence domain with health implications
Role of authenticity	Treated as a broad antecedent of trust	It is positioned as the primary explanatory construct.
Psychological mechanisms	Authenticity to the trust	Specific pathways through perceived sincerity, self-congruence, and emotional attachment
Theoretical integration	Single-theory	Implicit integration of SDT, SIT, and Self-Congruency Theory with attributed mechanisms
Mediating structure	Trust is frequently regarded as a direct indicator.	Authenticity is in fact linked to loyalty through trust which is the key mediator.
Moderators	Very few are mentioned explicitly	Theoretically, perceived health risk and product credence level are the most important boundary conditions.
Empirical approach	SEM or regression with limited process testing	SEM with bootstrapped indirect effects testing multi-step mediation
Managerial relevance	General trust-building directions	Nutrition brand actionable levers (evidence transparency, identity alignment, third-party verification)

The works, which have provided the most salient frameworks currently available on trust in a brand or the perception of authenticity, do so in the context of branding at large. There is, however, a distinct absence of applying those models to the nutrition industry. The psychological links that have been documented between brand trust, authenticity, and subsequent loyalty particularly associated with health-related consumer products, are rudimentary at best. This paper attempts to fill that gap with self-determination theory, social identity theory, and the cognitive-affective-behavioral model to provide a unique perspective on the nutrition branding psychology, and a more extensive base to build from in later research.

Building on the integrated mechanisms above, we propose that perceived sincerity and self-congruence would operate as the proximal mediators of authenticity’s effect on trust, while emotional attachment will emerge as a second-stage effective mediator linking trust to loyalty. Further, we expect two contextual moderators: (a) perceived health risk is higher perceived risk strengthens the cognitive value of self-congruity for uncertainty reduction; and (b) product credence level is higher credence amplifies the importance of transparent evidence and third-party verification for SDT-driven internalization.

Expanding the theoretical construct discussed in this paper, the empirical model proves brand authenticity the key variable, and perceived sincerity, self-congruence, and emotional attachment are the proximal variables that create consumer trust. The mediating variable that is operationalized as the means of connecting authenticity and brand loyalty is the consumer trust. The investigation of the above relationships is done through structural equation modelling where the subsequent parts explain the measurement validation, as well as the structural results.

## 3 Research methodology

The current study applies a quantitative, cross-sectional approach to understand the psychological dimensions of brand authenticity, trust, and loyalty in the context of nutrition branding. It attempts to shed light on the effects of authenticity on consumer trust and subsequently, brand loyalty. This section explains the research model, states the research aims and hypothesis, and describes data collection and analytic procedures.

### 3.1 Sampling and participants

This study is focused on consumers of nutrition and wellness products, particularly, and more broadly, users of protein supplements, organic food, and other health products. Participants were selected from the consumer population using sampling techniques so that key subgroups of nutrition product consumers were represented (15). Stratified sampling used three strata: (1) gender (male/female); (2) age group (18–24, 25–34, 35–44, 45+), and (3) purchase frequency of nutrition products (weekly, monthly, occasional). The final sample (*n* = 300) excess common SEM guidance and an *a priori* G*power check for medium effects (*f*^2^ = 0.15, *α* = 0.05, power = 0.80) indicated ~150–180 participants were sufficient; for CFA and SEM.

The study aimed for a target sample size of 300 participants to ensure sufficient power for SEM analysis. Participants were filtered based on their reported involvement in purchasing nutrition products to ensure relevance to the research questions.

This sample size provides adequate statistical power for SEM and mediation tests (G*Power analysis for medium effects, *f*^2^ = 0.15, *α* = 0.05, power = 0.8). Participants were screened for regular purchase of nutrition products to ensure relevance.

#### 3.1.1 Sample size determination

The final dataset consisted of 300 valid participants after screening and data cleaning. This sample meets recommended SEM sample guidelines. Second, using the 10-times rule for latent variable modeling, the final sample exceeds 10 times the number of indicators for the most complex construct in the model. Third, a-priori power analysis using G*Power suggested a minimum of 150–180 participants for detecting medium effects. Therefore, a sample of 300 provides adequate statistical power and estimation stability for CFA and SEM.

#### 3.1.2 Recruitment procedures

To recruit participants, we focused on a combination of online resources which are popular among nutrition- or health-concerned people. To be more exact, we added our sign-up links to BAND, KakaoStory Facebook and Instagram pages, focusing on health and fitness (such as nutrition, wellness, and fitness groups), placed them on Reddit forums that feature nutritious and healthy lifestyle content, as well as, placed them on popular health and wellness blogs and newsletters that publish evidence-based information on nutrition. The reasons we settled on these spaces was that this group of people is already interested in nutrition products and thus, the right audience to gauge the authenticity, trust, and loyalty in credence-based health markets. The information we gathered is that of consumers living in South Korea, hence the regulatory and cultural atmosphere of nutrition branding and health claims remains relatively similar. By remaining in a single country, we have eliminated much of the clatter of comparison between cultures when people reflect on the issues of authenticity and trust, which allows us to more readily observe the psychological matter that we want. Future research may extend the model to other geographies in future and observe the results of authenticity, trust and loyalty around the world.

Participants were eligible if they had purchased nutrition products within the past 3 months, ensuring that responses were relevant to the study context. An invitation to participate in the study was posted on these platforms with a link to the online survey. As an appreciation of their time and participation, we offered the participants some small gift cards. The survey was also structured to provide specific information on consumer attitude and behavior on brand authenticity, trust and loyalty in the nutritional sector. The self-administered online questionnaire was used to conduct it, whereby the respondent could complete the questionnaire at their convenience.

This recruitment strategy perfectly aligns with the theoretical concerns of the study, i.e., credence-based nutrition products, as individuals that are active in health-conscious Internet communities are more prone to the application of authenticity vibes and trust schemes when they go through nutrition brands.

#### 3.1.3 Response rate and demographic breakdown

The initial outreach targeted approximately 500 participants, resulting in 300 completed responses. The final response rate was therefore 60%. This response rate is considered acceptable for online surveys in this field and aligns with typical industry standards for consumers behavior studies. The sample demographics are summarized in Table 2.

**Table 2:** The sample demographics.

Demographic factor	Percentage (%)
Age	
18–24	25%
25–34	30%
35–44	20%
45+	25%
Gender	
Male	45%
Female	55%
Income	
<$30 K	35%
$30 K − $60 K	40%
>$60 K	25%
Frequency of purchase	
Weekly	30%
Monthly	40%
Occasionally	30%

To address potential social desirability bias (where participants may respond in ways they believe are socially acceptable rather than their true feelings), the survey was designed to ensure confidentiality and anonymity. Additionally, we included reverse-coded items in the trust and authenticity and anonymity. Additionally, we included reverse-coded items in the trust authenticity scales to help detect and control for socially desirable responding.

We also ensured that the wording of the survey questions was neutral and did not prompt any specific responses. A forced choice format was used for some questions to reduce the likelihood of participants choosing socially desirable options in a non-committal manner (16).

### 3.2 Confirmatory factor analysis (CFA)

CFA was conducted to evaluate the measurement model to verify that the constructs are well defined and the component constructs are reliably measured. Several indices of fit (Comparative Fit Index (CFI), the Tucker-Lewis Index (TLI), the Root Mean Square Error of Approximation (RMSEA) and the Standardized Root Mean Square Residual (SRMR)) were used to test the model fit.

### 3.3 Structural equation modeling (SEM)

SEM was used to measure the structural associations of authenticity, trust, and loyalty. It also examines their direct and indirect effects, particularly the mediating role of trust on the authenticity-loyalty relationship. All SEM analysis were performed on the validated measurement model, using standardized estimates and 5,000 bootstrap samples to test indirect effects.

SPSS and AMOS or SmartPLS were used for data analysis. The choice depends on the model specification and evaluation preference. Both CFA and SEM features offer the accuracy and reliability of the outcomes.

### 3.4 Testing for reliability and validity

Reliability and validity of all constructs were assessed using the current sample. Internal consistency was evaluated using Cronbach’s *α* and Composite Reliability (CR), with values above 0.70 indicating acceptable reliability. Convergent validity was assessed using standardized factor loadings and Average Variance Extracted (AVE), where AVE ≥ 0.50 indicates adequate convergence.

The data were analyzed using CFA and SEM to assess measurement validity, internal reliability, and the hypothesized structural relationships. CFA was conducted to elevate construct validity and reliability of the measurement model. SEM was then used to estimate the structural paths among authenticity, trust, and loyalty and to test mediation effects. Data analysis was performed using SPSS and AMOS (or SmartPLS where appropriate), depending on the specific model specifications and estimation requirements. Model fit was evaluated using the Chi-square (*x^2^*) and its ratio to degrees of freedom (*x^2^*/df), the CFI, TLI, RMSEA and SRMR. We used CFI/TLI ≥ 0.90 (preferably ≥0.95), RMSEA ≤ 0.06–0.08 (reporting 90% CI) and SRMR ≤ 0.08 as indicators of acceptable to good fit. Full reliability and validity indices (*α*, CR, AVE, and loadings) are presented in Table 3.

**Table 3:** Measurement constructs and items.

Construct	Measurement items
Authenticity	1. The brand is transparent in its practices.
	2. The brand maintains consistent messaging across all channels.
	3. The brand behaves ethically in all aspects of its operations.
	4. The brand genuinely cares about its customers’ well-being.
Trust	1. The brand is reliable.
	2. I trust the brand to deliver on its promises.
	3. The brand is honest in its advertising and communication.
	4. I believe the brand will act in my best interest.
Loyalty	1. I am committed to purchasing from this brand regularly.
	2. I prefer this brand over other competing brands.
	3. I would continue to purchase from this brand even if alternatives were available.
	4. I feel emotionally attached to this brand.

In this section, methods elaborates on an approach for examining the model that ties brand authenticity, brand trust, and brand loyalty together in the context of nutrition branding, and how they relate to each other (17). Using the survey approach, we identify the relations between the measures that are constructed and how brand trust and loyalty are influenced, while we utilize scales that have been validated.

The strengthened theoretical aspects of the nexus authenticity–trust–loyalty as well as the practical aspects of the methodology are how this is presented for nutrition brands to help them build closer relationships with their consumers. The literature gap we want to address is to provide more depth to the understanding of the psychology behind consumer behavior for the nutrition industry (18).

## 4 Results

The results are presented in two stages. First, we report the measurement model validation using CFA, including reliability (Cronbach’s *α*, CR), convergent validity (AVE), and discriminant validity criteria. Second, we report the SEM results used to test the hypothesized relationships among constructs. This two-step structure follows best practices for SEM-based studies and improves the coherence of the findings.

### 4.1 Sample characteristics

**Figure 2:**
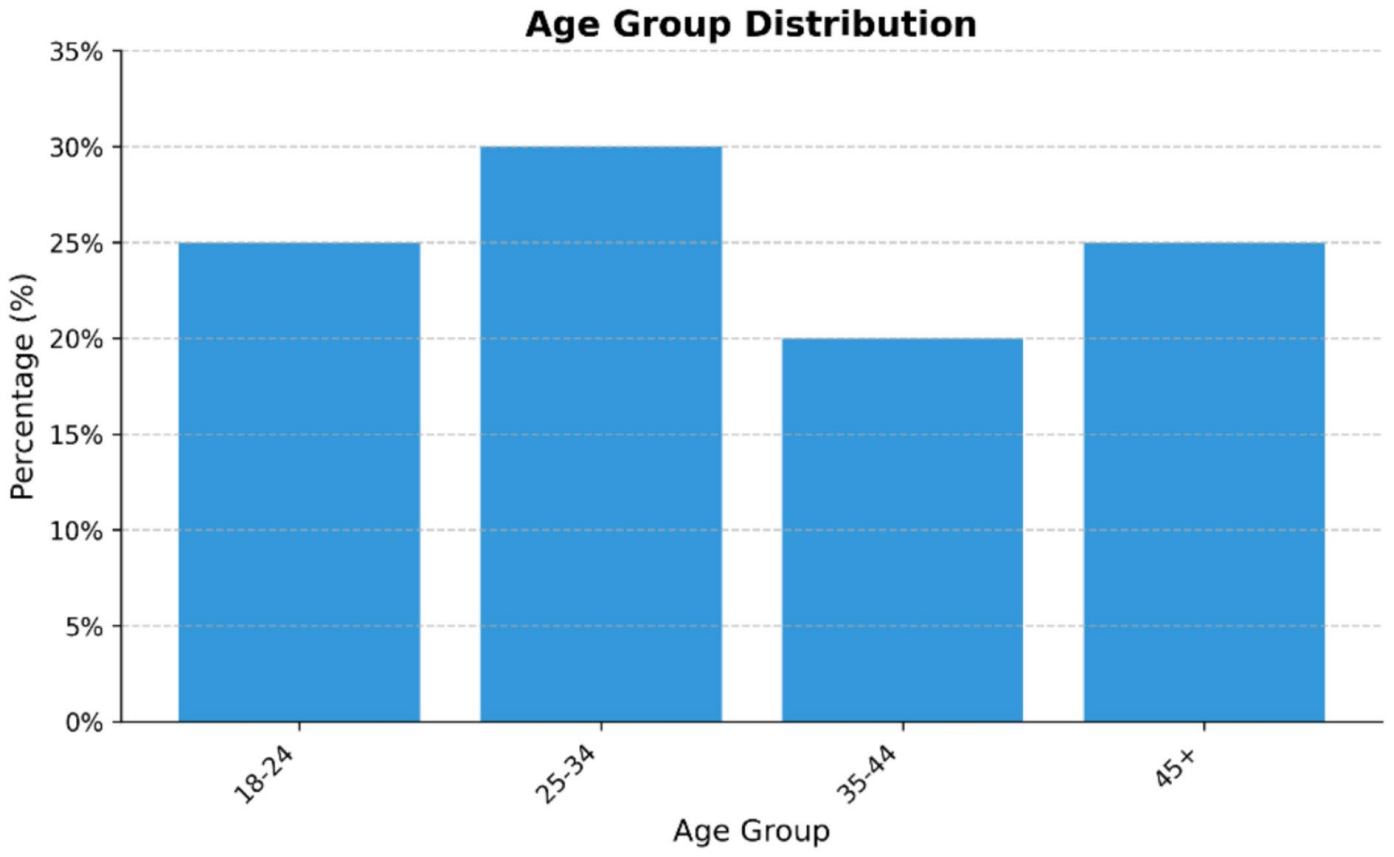
Age group distribution of respondents.

The final sample consisted of 300 respondents. Table 4 presents the demographic distribution with both frequencies (n) and percentages (%). Age groups were distributed as follows: 18–24 (25%), 25–34 (30%), 35–44 (20%), and 45 + (25%) (Figure 2). The gender distribution included 45% males and 55% females. The income categories were < 30 k (*n* = 105, 35%), 30 k-60 k (*n* = 120, 40%), and <60 k (*n* = 75, 25%). Regarding purchases frequency of nutrition products, 90 participants (30%) reported weekly purchases, 120 (40%) reported monthly purchases, 90 (30%) reported occasional purchases.

**Table 4:** Demographic summary of respondents.

Demographic factor	Percentage (%)	Frequency (n)
Age		
18–24	25%	75
25–34	30%	90
35–44	20%	60
45+	25%	75
Gender		
Male	45%	135
Female	55%	165
Income		
<$30 K	35%	105
$30 K − $60 K	40%	120
>$60 K	25%	75
Frequency of purchase		
Weekly	30%	90
Monthly	40%	120
Occasionally	30%	90

### 4.2 Measurement model (CFA)

CFA was conducted to assess reliability and validity of the constructs. As shown in Table 5, all standardized factor loadings exceeded the recommended threshold of 0.70. Cronbach’s *α* values ranged from 0.81–0.92, and Composite Reliability (CR) values ranged from 0.83–0.91, indicating strong internal consistency. Average Variance Extracted (AVE) values ranged from 0.56–0.68, exceeding the 0.5 criterion and confirming convergent validity.

**Table 5:** Measurement model results showing factor loading, internal consistency (Cronbach’s α), composite reliability (CR), and average variance extracted (AVE) for all constructs.

Construct	Items	Loading	Cronbach’s α	CR	AVE
Authenticity	A1	0.83	0.88	0.90	0.62
Authenticity	A2	0.80			
Authenticity	A3	0.79			
Perceived sincerity	PS1	0.77	0.84	0.86	0.56
Perceived sincerity	PS2	0.85			
Self-congruence	SC1	0.89	0.86	0.88	0.60
Trust	T1	0.88	0.92	0.91	0.68
Trust	T2	0.72			
Emotional attachment	EA1	0.84	0.81	0.83	0.56
Loyalty	L1	0.79	0.89	0.90	0.61
Loyalty	L2				

Discriminant validity was established using the Fornell-Larcker criterion, with the square root of each construct’s AVE exceeding the inter-construct correlations. Cronbach’s α, CR and AVE for each construct are reported in Table 4; α and CR ≥ 0.70 and AVE ≥ 0.50 were used a threshold for acceptable reliability and convergent validity (19).

### 4.3 Measurement model fit

The measurement model demonstrates satisfactory fit to the data:

*χ*^2^ = 312.45; *df* = 168; *χ*^2^/*df* = 1.86; CFI = 0.958; TLI = 0.947; RMSEA = 0.043 (90% CI 0.034, 0.052); SRMR = 0.041.

These fit indices indicate a good measurement model. CFI and TLI exceed conventional thresholds, RMSEA is below 0.05 indicating close fit, and SRMR is well under 0.08, supporting the adequacy of the measurement structure for subsequent structural tests.

These indices collectively demonstrate that the measurement model fits the data well. The CFI and TLI values recommended threshold of 0.90, indicating strong comparative fit. The RMSEA value of 0.043 is below the 0.05 criterion, suggesting a close fit of the model. These values meet commonly accepted SEM thresholds.

### 4.4 Structural model (SEM) results

The structural model also demonstrated acceptable fit. Authenticity had a strong positive effect on trust, and trust significantly predicted loyalty. The direct effect of authenticity on loyalty was small and non-significant, suggesting that the relationship operates mainly through trust. Other predictors (self-congruence, perceived sincerity, and emotional attachment) also showed meaningful positive effects. Overall, the structural paths support the proposed relationships.

**Figure 3:**
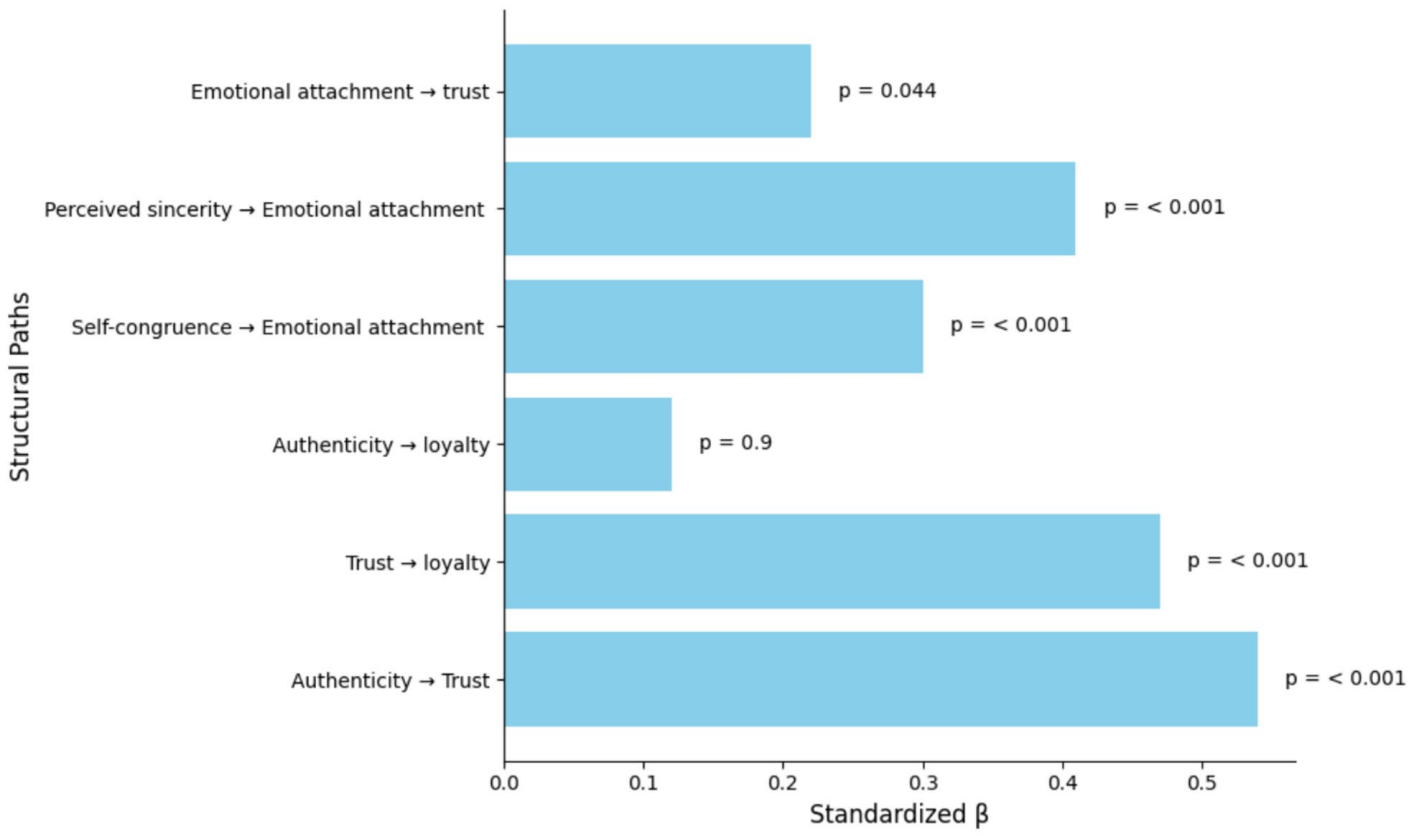
Standardized *β* values for structural paths in the model.

Perceived authenticity has a substantial positive effect on trust (*β* = 0.54, *p* < 0.001), and trust meaningfully predicts loyalty (*β* = 0.47, *p* < 0.001). Figure 3 shows the standardized *β* values for structural paths in the model. The small, non-significant direct path from authenticity to loyalty suggests that authenticity operates primarily through trust. Proximal mechanisms such as self-congruence and emotional attachment also meaningfully contribute to trust formation as shown in Table 6 and Figure 3.

**Table 6:** SEM model fit indices and standardized path coefficients.

Fit index	Value	Criterion	Interpretation
A. Model fit indices			
*χ* ^2^	345.78	—	Reported for completeness
df	170	—	—
*χ*^2^/df	2.03	< 3.0	Good fit
CFI	0.952	≥0.90	Good fit
TLI	0.943	≥ 0.90	Good fit
RMSEA	0.047	< 0.06	Good fit
RMSEA 90%	0.037, 0.056	<0.05	Acceptable
SRMR	0.045	< 0.08	Good fit

Path	Standardized *β*	*p*-value	Significance
B. Structural path estimates			
Authenticity → trust	0.54	< 0.001	Significance
Trust → loyalty	0.47	< 0.001	Significance
Authenticity → loyalty	0.12	0.90	Not significant
Self-congruence → emotional attachment	0.30	< 0.001	Significance
Perceived sincerity → emotional attachment	0.41	< 0.001	Significance
Emotional attachment → trust	0.22	0.044	Significance

Construct	(*R*^2^)	Interpretation
C. Explained variance (*R*^2^)		
Trust	0.58	Model explains 58% of trust
Loyalty	0.52	Model explains 52% of Loyalty

### 4.5 Mediation analysis

Bootstrapped mediation analysis indicated that trust significantly mediates the relationship between authenticity and loyalty. Additional indirect pathways through perceived sincerity, self-congruence, and emotional attachment were also significant. The bootstrapped indirect effect (authenticity → trust → loyalty) is significantly and the direct effect is non-significant, consistent with full mediation in this sample: authenticity increases loyalty primarily by increasing trust. The significance of indirect paths by sincerity, self-congruence and emotional attachment confirms these constructs act as proximal mechanisms as shown in Table 7.

**Table 7:** Structural model results.

Path	*β*	SE (boot)	*p*-value	95% CI
Authenticity → Trust	0.54	0.06	< 0.001	0.42, 0.66
Self-congruence → Trust	0.30	0.05	< 0.001	0.20, 0.40
Perceived sincerity → Emotional attachment	0.41	0.07	< 0.001	0.27, 0.55
Emotional attachment → Trust	0.22	0.07	0.004	0.07, 0.37
Trust → Loyalty	0.47	0.006	< 0.001	0.35, 0.59
Authenticity → loyalty (direct)	0.12	0.007	0.90	−0.02, 0.26
Authenticity → loyalty (indirect)	0.25	0.05	< 0.001	0.18, 0.33

Bootstrapped mediation results confirmed that trust significantly mediates the effect of authenticity on loyalty. The indirect effect was significant, while the direct effect was not, indicating that authenticity influences loyalty primarily through trust. Additional indirect effects by sincerity, self-congruence, and emotional attachment were also supported.

### 4.6 Hypothesis testing summary

Taken together, the hypothesis tests show a coherent pattern: H1 and H2 are supported (authenticity → trust; trust → loyalty), H3 is supported by a significant bootstrapped indirect effect, and H4 is supported as perceived sincerity, self-congruence, and emotional attachment act as significant proximal mediators. The model explains a substantial share of variance in the endogenous variables (*R*^2^ = 0.58 for Trust; *R*^2^ = 0.52 for Loyalty), indicating that the proposed predictors account for a meaningful portion of why consumers come to trust and remain loyal to nutrition brands. These results provide both statistical and practical evidence that interventions designed to increase perceived authenticity (e.g., transparent sourcing, evidence-based claims, identity-congruent messaging) are likely to have downstream benefits for trust primarily by strengthening the mediating beliefs and feelings that make consumers rely on the brand. These empirical results form the basis for the discussion that follows, where we compare our findings to prior studies and derive managerial implications.

## 5 Discussion

This study examined how brand authenticity affects consumer trust and loyalty in nutrition branding. The findings of this study reinforce the central role of brand authenticity in shaping consumer evaluations within nutrition markets; a domain characterized by information asymmetry and elevated perceived health risk. The structural model confirmed that authenticity has a strong and significantly positive influence on trust (*β* = 0.54, *p* < 0.001), consistent with Self-Determination Theory, which argues that perceptions of sincerity and transparency facilitate internalization of brand values. Trust, in turn, emerged as a powerful driver of loyalty (*β* = 0.47, *p* = 0.001), supporting prior work that positions trust as the principal mechanism through which consumers navigate credence-based product claims. Therefore, the direct correlation between authenticity and loyalty actually did not emerge but in the trust factor, it completely mediates. That demonstrates that authenticity is the primary driver with regard to the actions of consumers. The mediating variables of perceived sincerity, self-congruence, and emotional attachment are also determined to be acted in combination with authenticity, such as self-brand fit, credibility signals, bonding, attachment. This is what previous studies on consumer behavior show, and this simply indicates that when people perceive a nutrition brand as authentic, transparent, and consistent with their own principles, they tend to believe in it more and adhere to it over the long-term.

### 5.1 Psychological interpretations of the findings

The findings of this study can be analyzed, explained, and fully understood through certain established psychological models that can further illuminate the processes through which trust and loyalty are tied to brand authenticity.

### 5.2 Self-determination theory (SDT)

In the light of SDT, the results indicate that authenticity satisfies consumers’ intrinsic need for genuineness and self-alignment. Individuals, SDT posits, are encouraged to pursue activities that are consistent with their values and beliefs, especially when making decisions about their health and wellness. To many consumers, authentic brands are the epitome of honesty, transparency, and alignment with personal values. This intrinsic alignment leads to a more positive consumer sentiment and trust, as consumers believe that there is a brand that resonates with their identity and value system. This theoretical prediction is empirically supported by significant positive effect of self-congruence on trust (*β* = 0.30, *p* < 0.001), which means that when consumers believes that a nutrition brand as a part of their own values and health objectives, they become more prone to internalize the intentions of the brand and turn to it as a reliable source. This result is a direct consequence of the statement of SDT that value internalization and autonomous motivation reinforced trust formation, especially in credence-based health situations.

Consumers appreciate the absence of brands short-term focus, and recognize the desire to make a meaningful difference in their lives, addressing their psychological need for autonomy and self-expression. Nutrition brands, in this case, are able to use the authenticity cues to incentivize consumer engagement, thereby fostering stronger emotional connections with the brand.

### 5.3 Social identity theory (SIT)

SIT, or Social Identity Theory, serves as another useful lens for explaining the relationships that we have seen. Its theories that people form part of their self-concept from the groups that they associate with and often attempt to have positive association with brands that support their social identity. This includes people who associate with certain attitudes and practices such as personal health, organic products, or holistic health. These people within these communities under health and wellness are called health-conscious consumers. These brands that are authentic to their practices and purpose help to amplify this association. These consumers actually relate with a nutrition brand at a personal level when they consider it. Such an emotional response facilitates the trust to the brand since they perceive the brand as something community-based and hence trustworthy. The Social Identity Theory is supported by our research, as it demonstrates that real emotional relationships with consumers can be established with the help of authentic brands. These relationships are what help to maintain trust, which consequently increases the brand loyalty. Based on the Social Identity Theory, the data indicate that trust building conditions indeed depend on emotional attachment (*β* = 0.22, *p* = 0.004), that is, identity alignment and sense of belonging are critical when consumers evaluate the credibility of nutrition brands. This supports SIT’s claim that brands acting as social and identity symbols build trust by reinforcing group membership and social validation, which subsequently promotes the outcomes associated with loyalty.

### 5.4 Cognitive-affective-behavioral model

The Cognitive-Affective-Behavioral model describes a scenario in which the trust and loyalty which stems from authenticity stems from a fundamental reworking of the idea of a brand and how reliable and honest it is perceived to be. Authenticity makes a brand reliable in the user’s eyes, which is a cognitive assessment. This, in turn, leads to Affective responses, trust and emotional attachment. Behaviorally, trust is the primary reason repeat purchasing and brand loyalty is exhibited. The data show that when people believe a brand is authentic, they are more likely to trust it (*β* = 0.54, *p* < 0.001). That trust, in turn, predicts higher brand loyalty (*β* = 0.47, *p* < 0.001). The combination of these results provides direct support to the cognitive affective behavioral chain that the model suggests, wherein cognitive appraisal of authenticity triggers affective trust, which consequently converts into consumer intentions of loyalty in nutrition markets.

This study demonstrates that authenticity does ‘activate’ the cognitive-affective-behavioral model, and illustrates how consumer brand trust is generated and through that, loyalty. This model emphasizes the psychological shift that a consumer makes, in order to shed light on the rationale behind loyalty to genuine brands and how it deepens the consumer’s attachment to the brand.

### 5.5 Comparison to previous research

Our results align with a growing literature documenting positive links between brand authenticity, trust, and loyalty. We acknowledge substantial overlap with these studies in documenting authenticity as an antecedent of trust and loyalty. However, the present research offers three clarifying extensions. First, by mapping SDT, SIT, and Self-Congruity to specific mechanisms and testing them jointly, we provide evidence about which psychological pathways dominate in a nutrition (credence) context, whereas much prior work treated authenticity as a single undifferentiated predictor. Second, our mediation tests (SEM with bootstrapped indirect effects) clarify that authenticity’s effect on loyalty operates primarily by trust, with emotional attachment and perceived sincerity as proximal mediators, a more nuanced processed than simple bivariate associations. Third, by explicitly discussing boundary conditions (perceived health risk and product credence), we identify conditions under which the relative importance of cognitive versus affective mechanisms shifts. These contributions should be read as incremental but theory-enhancing refinements to existing consumer behavior findings rather than claims of complete novelty. A positive link between trust and loyalty in general consumer settings. Our findings are consistent with this relationship: trust strongly predicted loyalty (*β* = 0.47, *p* < 0.001). However, unlike their broad consumer samples, we examine a nutrition/ credence context. In such contexts, trust appears to act as a more central mediator, absorbing much of authenticity’s influence on loyalty, a pattern likely attributable to increased uncertainty and risk in health-related purchases. That authenticity predicts satisfaction and repeat purchases intentions in online retail contexts. We replicate the broad predictive the broad predictive role of authenticity, but extend their findings by decomposing the mechanisms: perceived sincerity and self-congruence emerge as proximal mediators in our model, and emotional attachment provides an affective pathway that supports trust. This suggests that the psychological process is more nuanced in nutrition markets.

Similarly, SDT-based processes that prior research has found essentially imply that it is the internalization of values that leads to continued consistent action in people (20). Our findings agree with that thin-view concept, the idea of authenticity does its work on trust because we internalize how honest we feel and whether that resonates with our own self-concept, and this is what SDT predicts. This similarity only goes to indicate how handy it is to combine motivational material with identity material when we are attempting to describe brand behavior in health situations (21).

### 5.6 Theoretical implications

The findings offer clear, theory-grounded guidance for managers in nutrition and health-related markets. First, it has a great impact on the perceived authenticity and trust (*β* = 0.54, *p* < 0.001) demonstrating that the transparency is indeed worth it. According to the Self-Determination Theory, such aspects as transparent ingredient labeling, third-party testing, evidence-based health claims assist customers internalize value and as a result, consumers can develop autonomous trust rather than simply pursuing price reductions.

Second, the statistically significant correlation between self-congruence and trust (*β* = 0.30, *p* < 0.001) indicates the importance of identity-relevant messaging. According to the Social Identity Theory, the nutrition brands are able to increase perceived self-brand fit through the construction of a narrative that conforms to the health objectives and health lifestyles of the consumers, such as the fitness-oriented or environmentally friendly roles, creating the trust by means of social validation.

Third, the role of emotional attachment in trust (*β* = 0.22, *p* = 0.004) indicates that authentic storytelling, involvement in the community, and personalized communication are actually important. These affective strategies are accompanied by the cognitive aspect of investigating authenticity and enhancing trust in the cognitive-affective-behavioral model.

Finally, trust actually makes a significant difference in loyalty (*β* = 0.47, *p* < 0.001) revealing that this factor is the primary factor which makes authenticity translate to brand commitment. Authenticity should therefore be regarded by managers as a long-term approach to bind transparency, identity alignment, and emotional involvement to gain sustained loyalty on credence-based nutrition markets.

## 6 Conclusion and further research directions

This study examined how perceived brand authenticity shapes consumer trust and loyalty in nutrition markets; a credence domain characterized by information asymmetry and heightened perceived health risk. Using survey data from 300 consumers and validated measurement and structural models (CFA and SEM with bootstrapped indirect tests), we found that perceived authenticity substantially increases consumer trust (*β* = 0.54, *p* < 0.001), and that trust in turn strongly predicts loyalty (*β* = 0.47, *p* < 0.001).

Theoretically, these findings refine understanding of authenticity by mapping distinct psychological mechanisms to established frameworks (Self-Determination Theory, Social Identity Theory, and Self-Congruity Theory) and demonstrating their joint operation in a credence context. Practically, results imply that nutrition brands seeking durable consumer loyalty should prioritize actions that enhance perceived authenticity and strengthen the mediators identified here: transparent product information and sourcing (to raise perceived sincerity), identity-congruence messaging (to improve self-congruence), and initiatives that build emotional attachment (brand communities, storytelling). Taken together, the study offers both an empirically supported process model and concrete managerial levers for strengthening trust and loyalty in nutrition markets.

### 6.1 Theoretical contributions

This study refines existing understandings of brand authenticity by assigning distinct cognitive and affective mechanisms to established theories and demonstrating how those mechanisms operate in a nutrition context. Instead of making a new statement about the importance of building connections between authenticity, trust and loyalty, we introduce a specific, empirically tested theoretical framework that explains mediating processes, the context of health and boundary conditions related to risk and credence. These small steps forward, which we expect to make in the future, will guide further, more incremental studies in the fields of nutrition and other credence areas.

This approach position grants a more comprehensive understanding of how authenticity plays out in the processes of consumer-brand relational relationships. SDT explains how authenticity will fulfil the intrinsic consumer necessity of genuineness and self-congruency, whereas SIT highlights the central role of authentic brands in group identification and emotional connection on the part of health-oriented consumers. All these theoretical models taken together help us in understanding more about trust and loyalty in health-related consumer behavior, especially the underlying psychological processes.

Although these findings are robust in the current sample, they are based on cross-sectional survey data and modeled associations; causal claims should be made cautiously and future research should replicate these mechanisms using longitudinal or experimental designs.

### 6.2 Study limitations

This study has several limitations that provide direction for future work. First, the cross-sectional design restricts causal inference, as the relationships among authenticity, mediators, trust, and loyalty were tested at a single point in time. Second, the use of self-reported survey data introduces the risk of common-method bias and may not fully capture actual consumer behavior. Third, although the sample size of 300 was adequate for SEM, it relied on an online panel composed primarily of consumers already familiar with nutrition products, which may limit generalizability to broader or less-engaged populations. Fourth, the study examined a specific of mediators perceived sincerity, self-congruence, and emotional attachment while other potential psychological mechanisms (e.g., perceived risk reduction, value congruence, and health motivation) were not included. Finally, the model was tested within a single national context, which may not reflect cultural differences in authenticity perceptions or trust formation. These constraints should be addressed in future studies using longitudinal, experimental, behavioral, or cross-cultural approaches.

### 6.3 Future research

Future research should build on this study by employing longitudinal or experimental designs to establish causal ordering among authenticity, mediating mechanisms, trust and loyalty, and by examining boundary conditions such as perceived health risk, product type, consumer expertise, and cultural context to understand when cognitive or effective pathways dominate. Future work should incorporate behavioral measures such as purchase data or renewal behavior to validate whether attitudinal loyalty translates into actual consumer actions. Experimental studies testing authenticity-enhancing interventions including transparent labeling, third-party certification, and identity-based messaging would extend managerial relevance by estimating causal effects. Additionally, segment-based analysis could identify whether different consumer groups rely on distinct authenticity cues, while cross-channel comparisons could clarfy how platform characteristics shape credibility and trust formation. Mixed-methods research, combining surveys with interviews or ethnographic insights, would also help uncover nuanced perceptions of authenticity and reveal new mechanisms not captured by the present model.

## Data Availability

The raw data supporting the conclusions of this article will be made available by the authors, without undue reservation.

## References

[1] Aaker DA. Managing brand equity: capitalizing on the value of a brand name Simon and Schuster (2009).

[2] Huang D Markovitch DG Stough RA. Can chatbot customer service match human service agents on customer satisfaction? An investigation in the role of trust. J Retail Consum Serv. (2024) 76:103600. doi: 10.1016/j.jretconser.2023.103600

[3] Jian Y Zhou Z Zhou N. Brand cultural symbolism, brand authenticity, and consumer well-being: the moderating role of cultural involvement. J Prod Brand Manag. (2019) 28:529–39. doi: 10.1108/JPBM-08-2018-1981

[4] Singh C Dash MK Sahu R Kumar A. Investigating the acceptance intentions of online shopping assistants in E-commerce interactions: mediating role of trust and effects of consumer demographics. Heliyon. (2024) 10:e25031. doi: 10.1016/j.heliyon.2024.e25031, 38318071 PMC10840014

[5] Wu Y Zhu W. The role of CSR engagement in customer-company identification and behavioral intention during the COVID-19 pandemic. Front Psychol. (2021) 12:721410. doi: 10.3389/FPSYG.2021.721410, 34475843 PMC8407001

[6] Boekaerts M Maes S Karoly P. Self-regulation across domains of applied psychology: is there an emerging consensus? Appl Psychol. (2005) 54:264–265. doi: 10.1111/j.1464-0597.2005.00201.x

[7] Hung H-Y. Attachment, identification, and loyalty: examining mediating mechanisms across brand and brand community contexts. J Brand Manag. (2014) 21:594–614. doi: 10.1057/bm.2014.30

[8] Adegbeye MJ Elghandour MMMY Barbabosa-Pliego A Monroy JC Mellado M Ravi Kanth Reddy P . Nanoparticles in equine nutrition: mechanism of action and application as feed additives. J Equine Vet Sci. (2019) 78:29. doi: 10.1016/j.jevs.2019.04.001, 31203981

[9] Li C Chen H. Cultural psychology of English translation through computer vision-based robotic interpretation. Learn Motiv. (2023) 84:101938. doi: 10.1016/j.lmot.2023.101938

[10] Tam KP Milfont TL. Towards cross-cultural environmental psychology: a state-of-the-art review and recommendations. J Environ Psychol. (2020) 71:101474. doi: 10.1016/J.JENVP.2020.101474

[11] Wylleman P. An organizational perspective on applied sport psychology in elite sport. Psychol Sport Exerc. (2019) 42:89–99. doi: 10.1016/j.psychsport.2019.01.008

[12] Sattorov A. Emotional appeal as a determinant of effectiveness in English advertising texts: a linguistic and cognitive analysis. Int J Ind Eng Technol Operations Manag. (2024) 2:71–7. doi: 10.62157/ijietom.v2i2.64

[13] Van Dooren C Marinussen M Blonk H Aiking H Vellinga P. Exploring dietary guidelines based on ecological and nutritional values: a comparison of six dietary patterns. Food Policy. (2014) 44:36–46. doi: 10.1016/j.foodpol.2013.11.002

[14] Heckert J Martinez EM Sanou A Pedehombga A Ganaba R Gelli A. Can a gender-sensitive integrated poultry value chain and nutrition intervention increase women’s empowerment among the rural poor in Burkina Faso? J Rural Stud. (2023) 100:103026. doi: 10.1016/j.jrurstud.2023.103026, 37377776 PMC10291270

[15] Steinmuller PL Kruskall LJ Karpinski CA Manore MM Macedonio MA Meyer NL. Academy of nutrition and dietetics: revised 2014 standards of practice and standards of professional performance for registered dietitian nutritionists (competent, proficient, and expert) in sports nutrition and dietetics. J Acad Nutr Diet. (2014) 114:631–641.e43. doi: 10.1016/j.jand.2013.12.021, 24656504

[16] Waxman A. Prevention of chronic diseases: WHO global strategy on diet, physical activity and health. Food Nutr Bull. (2003) 24:281–4. doi: 10.1177/156482650302400307, 14564933

[17] Palazzolo M Pattabhiramaiah A. The minimum wage and consumer nutrition. J Mark Res. (2021) 58:845–69. doi: 10.1177/00222437211023475

[18] Hawkes C. The role of foreign direct investment in the nutrition transition. Public Health Nutr. (2005) 8:357–65. doi: 10.1079/PHN2004706, 15975180

[19] Cheung GW Cooper-Thomas HD Lau RS Wang LC. Reporting reliability, convergent and discriminant validity with structural equation modeling: a review and best-practice recommendations. Asia Pac J Manag. (2024) 41:745–83. doi: 10.1007/s10490-023-09871-y

[20] Alberts L Lyngs U Lukoff K. Designing for sustained motivation: a review of self-determination theory in behaviour change technologies. Interact Comput. (2024):iwae040. doi: 10.1093/iwc/iwae040

[21] Barkat H Riaz B Fatima A AlShammari L Ahmed YB Ahmad W . Nutritional, medicinal, and commercial significance of *Moringa oleifera* L. leaves: a comprehensive review. Chem Biodivers. (2025) 22:e202500559. doi: 10.1002/cbdv.202500559, 40195685

